# Crocetin Overproduction in Engineered *Saccharomyces cerevisiae* via Tuning Key Enzymes Coupled With Precursor Engineering

**DOI:** 10.3389/fbioe.2020.578005

**Published:** 2020-09-04

**Authors:** Tianqing Song, Nan Wu, Chen Wang, Ying Wang, Fenghua Chai, Mingzhu Ding, Xia Li, Mingdong Yao, Wenhai Xiao, Yingjin Yuan

**Affiliations:** ^1^Frontier Science Center for Synthetic Biology, Key Laboratory of Systems Bioengineering (Ministry of Education), School of Chemical Engineering and Technology, Tianjin University, Tianjin, China; ^2^Collaborative Innovation Center of Chemical Science and Engineering (Tianjin), Tianjin University, Tianjin, China

**Keywords:** metabolic engineering, crocetin, enzyme fusion, precursor engineering, *Saccharomyces cerevisiae*

## Abstract

Crocetin, an important natural carotenoid dicarboxylic acid with high pharmaceutical values, has been successfully generated from glucose by engineered *Saccharomyces cerevisiae* in our previous study. Here, a systematic optimization was executed for crocetin overproduction in yeast. The effects of precursor enhancement on crocetin production were investigated by blocking the genes involved in glyoxylate cycle [citric acid synthase (*CIT2*) and malic acid synthase (*MLS1*)]. Crocetin titer was promoted by 50% by Δ*CIT2* compared to that of the starting strain. Then, the crocetin production was further increased by 44% through introducing the forward fusion enzymes of *Ps*CrtZ (CrtZ from *Pantoea stewartii*)-*Cs*CCD2 (CCD2 from *Crocus sativus*). Consequently, the crocetin titer reached to 1.95 ± 0.23 mg/L by overexpression of *Ps*CrtZ-*Cs*CCD2 followed by medium optimization. Eventually, a titer of 12.43 ± 0.62 mg/L crocetin was achieved in 5-L bioreactor, which is the highest crocetin titer reported in micro-organisms.

## Introduction

Crocetin, a carotenoid derivative compound isolated from the stigma of *Crocus sativus* L (Saffron), has great potential in the clinical treatment of cardiovascular and neuropsychological diseases (such as hypertension, atherosclerosis, and depression) (Nassiri-Asl and Hosseinzadeh, 2015; Xiang et al., 2017), and even in cancer therapy (Bathaie et al., 2013; Moradzadeh et al., 2018). Due to the limitations of saffron resources and the lower yield of traditional extraction, the output of crocetin is far from the market demand. *De novo* biosynthesis of crocetin in yeast (Chai et al., 2017) and *Escherichia coli* (Wang et al., 2019) has been achieved, respectively, but the titers were still quite low. Learning from previous results, two main clues for further optimization are enriching precursor pools and tuning limited metabolic reaction step (conversion zeaxanthin to crocetin dialdehyde by CCD).

**FIGURE 1:**
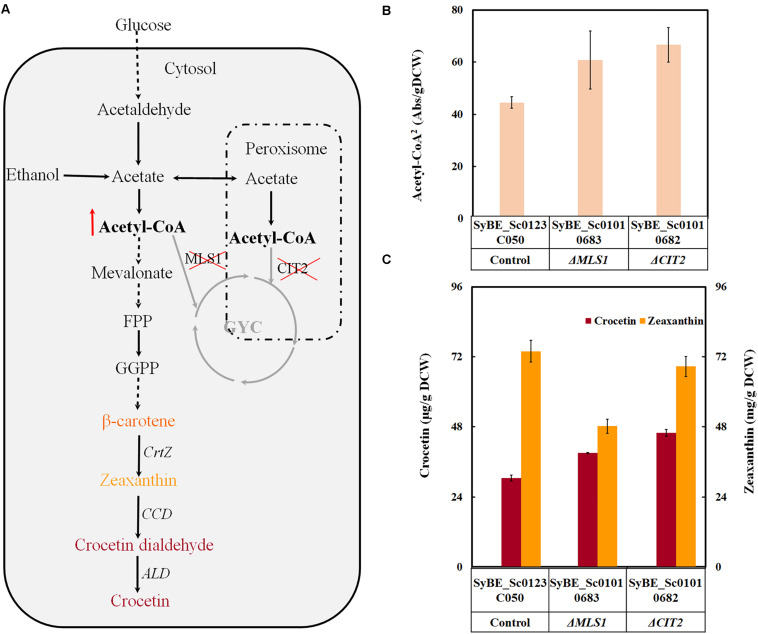
The effect of precursor engineering on crocetin output in *S. cerevisiae*. **(A)** The paradigm of crocetin biosynthetic pathway optimization by precursor engineering in *S. cerevisiae*; **(B)** The relative acetyl-CoA concentration per gDCW in the gene knockout (Δ*CIT2* and Δ*MLS*) strains; **(C)** The effects of gene knockout on crocetin and zeaxanthin output. The production of crocetin and zeaxanthin was quantified by μg/gDCW. The error bars represent standard deviations calculated from triplicate experiments.

On one hand, the *de novo* crocetin biosynthesis pathway in *Saccharomyces cerevisiae* is relatively long and consecutive steps comprised the mevalonate pathway (MVA) as precursor pool and the following heterologous crocetin formation pathway (Figure 1A). Thus, to enhance the MVA pathway flux is also crucial for final crocetin production. Previous efforts to engineer MVA pathway relevant genes to increase acetyl-CoA and MVA levels have achieved significant success in boosting terpenoid yield and productivity in *S. cerevisiae* (Ro et al., 2006; Lv et al., 2016). At the same time, it should be noted that acetyl-CoA used for terpenoids production is in the cytoplasm. Acetyl-CoA in the peroxisomes and cytosol could be converted to C_4_ organic acids (malate and succinate) via the glyoxylate cycle (GYC) (Figure 1A), that will be transferred to the mitochondria for oxidation by malic enzyme and the tricarboxylic acid (TCA) cycle (Chen et al., 2013). Previous studies had revealed that deleting the related proteins of the GYC [i.e., citric acid synthase (CIT2) in peroxidase body and malic acid synthase (MLS1) in the cytoplasm] was to decrease the digestion of acetyl-CoA in the cytoplasm to be used for the TCA cycle (Cobine et al., 2012; Chen et al., 2013; Krivoruchko et al., 2015). Thus, in order to further enhance precursor pools, deleting related genes to avoid acetyl-CoA entering GYC cycle and to prevent FPP consumption by dephosphorylating would be a good choice.

On the other hand, efforts in protein fusion formed by linker have been widely used to promote heterologous production of versatile products in micro-organisms (Deng et al., 2016). Zhou et al. (2012) reported that miltiradiene production was significantly improved and byproduct accumulation was reduced by the fusion of copalyl diphosphate synthase (*Sm*) and kaurene synthase-like (*Sm*KSL) from *Salvia miltiorrhiza*, as well as the fusion of GGPP synthase (BTS1) and farnesyl diphosphate synthase (ERG20). In addition to linker type (Sabourin et al., 2007) and linker length (Zhao et al., 2016), fusion orientation (forward or reverse) could significantly affect the performance of fusion protein. Lee et al. (2016) pointed out fusing a coumarate-CoA ligase (4CL) and benzalace-tone synthase from *Rheum palmatum* (*Rp*BAS) in the 4CL-*Rp*BAS orientation gave significant improvement on final raspberry ketone levels, but the reverse version (*Rp*BAS-4CL) did not work. In our previous work, it was found that the fusion orientation of geraniol synthase (GES) and ERG20 provided no significant effects on geraniol production in *S. cerevisiae* (Jiang et al., 2017). In our current case, since CCD2 has a more hydrophobic substrate channel on N-terminal (Chai et al., 2017), and CrtZ is just responsible for zeaxanthin formation (which is the substrate of CCD2), the fusion of CrtZ and CCD2 would enhance the substrate accessibility of CCD2, consequently leading to higher zeaxanthin conversion. And because the previously available structural study of CCDs revealed that a striking “windmill” architecture consisting of seven-bladed β-propeller was highly conserved on the C-terminal, thus it is also worth to explore the fusion orientation of CrtZ and CCD2 in the following work for further promoting crocetin level.

In present work, the crocetin production was further improved by precursor engineering coupled with tuning key enzymes. Firstly, two genes *CIT2* and *MLS1* that consuming cytoplasm acetyl-CoA were individually knocked out. The corresponding results demonstrated that crocetin titer was enhanced by 50% with *CIT2* knockout. Secondly, a synthetic fusion protein of CrtZ and CCD2 was constructed for unlocking the limiting step and the effect of fusion protein orientation was also investigated. Protein structural simulation revealed that the fusion orientation would have significant influence in the catalytical activity of fusion protein of CrtZ and CCD2. And the forward fusion enzymes of *Ps*CrtZ-*Cs*CCD2 promoted the crocetin titer by 44%, which just matched the protein structural simulation results. Finally, a titer of 12.43 ± 0.62 mg/L crocetin was obtained by medium optimization and 5-L fed-batch fermentation, which was the highest crocetin titer reported in micro-organisms so far. This study not only completely reproduces the classic metabolic engineering research loop from precursor engineering to enzyme engineering and process optimization, but also highlights the synergy between precursor engineering and key enzymes engineering is important to achieve microbial overproduction of natural products.

## Materials and Methods

### Strains Cultivation

All the yeast strains used in this study were listed in Table 1. For shake-flask cultivation, colonies were picked from solid plates and cultured in 3 mL SC medium (Chai et al., 2017) for overnight growth at 30°C. Then 500 μL culture was transferred into 5 mL fresh SC medium for seeds growing. After nearly 15 h cultivation, the seed culture was inoculated into 50 mL YPD medium with an initial OD_600_ of 0.1. For crocetin production, 1% (w/v) D-(+)-galactose was supplied into YPD medium as the inducer. The whole fermentation lasted for 96 h at 20°C. In the meanwhile, samples were collected at 42 h and the concentration of acetyl-CoA is quantified by BioVision’s PicoProbe Acetyl CoA Assay Kit (MAK039), according to the protocols provided by the manufacturer.

**TABLE 1:** *S. cerevisiae* strains used in this study.

Strain	Description	Source
SyBE_Sc0014CY06	β-carotene producing strain	This lab
SyBE_Sc0123C050	SyBE_Sc0014CY06, Δ*ho:*P*_GAL__1_-PsCrtZ-*T*_*HIS*__5_*, pRS416*-*T*_*HIS*__5_-*P*_GAL__10_-CsCCD2-*T*_*TEF*__2_-*P*_GAL__7_-SynALD*ALD*-*T*_PGI__1_*	This lab
SyBE_Sc01010682	SyBE_Sc0123C050, Δ*CIT2*	This study
SyBE_Sc01010683	SyBE_Sc0123C050, Δ*MLS1*	This study
SyBE_Sc02130062	SyBE_Sc0014CY06, Δ*CIT2*, pRS416-P*_GAL__10_*-*PsCrtZ*–*CsCCD2*-T*_*TEF*__2_*-P*_GAL__7_*-*SynALD*-T*_PGI__1_*^1^	This study
SyBE_Sc02130063	SyBE_Sc0014CY06, Δ*CIT2*, pRS416-P*_GAL__10_*-*CsCCD2*–*PsCrtZ*-T*_*TEF*__2_*-P*_GAL__7_*-*SynALD*-T*_PGI__1_*^1^	This study
SyBE_Sc02130068	SyBE_Sc0014CY06, Δ*CIT2*, pRS426-P*_GAL__10_*-*PsCrtZ*–*CsCCD2*-T*_*TEF*__2_*-P*_GAL__7_*-*SynALD*-T*_PGI__1_*^1^	This study

*^1^– is for protein fusion.*

### DNA Manipulation and Strain Construction

All the plasmids (listed in Supplementary Table S1) used in this study were constructed in *E. coli* DH5α. All the primers used in this study were presented in Supplementary Table S2. The gene knockout strains were constructed by yeast homologous recombination method. The basic gene knockout cassette was designed as left homologous arm (gene_L)-*URA3*-right homologous arm (gene_R) and was assembled by overlap extension PCR (OE-PCR). Among these three parts, the left or right homologous arms were obtained by PCR amplification based on the sequences of *CIT2*, or *MLS1* in *Saccharomyces* Genome Database (SGD). Finally, the gene knockout cassette (gene_L-*URA3*-gen_R) was cloned into plasmid pEASY-Blunt (generating plasmids pEASY-Blunt-Q-03,04) for sequencing verification. Yeast transformation was conducted *via* the LiAc/SS carrier DNA/PEG method (Gietz, 2006). Before that, these plasmids should be digested with *Not*I. The recombinant yeasts were selected on SC-URA (Chai et al., 2017) plates and verified by PCR. Genome integration marker *URA3* was recycled through 5-FOA selection according to Boeke et al. (1984).

CrtZ and CCD2 were fused with the short flexible GGGS linker (Yu et al., 2015). In order to construct protein fusion cassettes, the promoters (P*_GAL__1_*, P*_GAL__7_*, and P*_GAL__10_*), and terminators (T*_*HIS*__5_*, T*_*TEF*__2_*, and T*_PGL__1_*) were amplified from the genomic DNA of *S. cerevisiae* CEN.PK2-1C. *PsCrtZ* and *CsCCD2* were recovered from plasmid pUC57-Simple-05 and pUC57-Simple-10 *via* PCR amplification, respectively. Cassette P*_GAL__10_*-*PsCrtZ*–*CsCCD2*-T*_*TEF*__2_*-P*_GAL__7_-SynALD*-T*_PGI__1_* and P*_GAL__10_*-*CsCCD2*–*PsCrtZ*-T*_*TEF*__2_*-P*_GAL__7_-SynALD*-T*_PGI__1_* (for protein fusion) were assembled by OE-PCR. The OE-PCR products were digested by *Bam*HI/*Eco*RI and inserted into the corresponding sites of plasmid pRS416, obtaining the plasmid pRS416-L-04 and pRS416-L-05, respectively (Supplementary Table S1). Then the module P*_GAL__10_*-*PsCrtZ-CsCCD2*-T*_*TEF*__2_-*P*_GAL__7_-SynALD-*T*_PGI__1_* was cut from pRS416-L-04 by *Bam*HI/*Eco*RI and inserted into the same site of plasmid pRS426, generating plasmid pRS426-L-01 (Supplementary Table S1). Transformation of plasmids into yeast strains was performed with the same method for gene knockout.

### Homology Modeling and Structural Analysis by Computational Simulation

To explore the effect of protein fusion version of CrtZ and CCD2, the tridimensional structure models of the *Cs*CCD2, forward fusion-protein *Ps*CrtZ-*Cs*CCD2 and reverse fusion-protein *Cs*CCD2-*Ps*CrtZ were built and optimized by EasyModeller (Kuntal et al., 2010). Two reported structures from PDB (PBDID: 4zri and 3npe) were used as the templates of *Ps*CrtZ and *Cs*CCD2, respectively. The three modeled structures were analyzed using Pymol software (Delano and Bioinformatics, 2002).

### Medium Optimization at Shake Flaks Level

Plackett-Burman (Ahuja et al., 2004) two-level fractional factorial design was used to verify the main effects among the conventional parameters including carbon source, nitrogen source and inorganic salt ions associated with yeast growth and crocetin production. *N* = 12 experimental design was selected to explore the significance of seven factors including glucose, yeast extract, peptone, (NH_4_)_2_SO_4_, MgSO_4_, KH_2_PO_4_, and CaCl_2_. Two levels’ design was set for each factor, as shown in Supplementary Tables S3, S4. Then, the designed experiment was performed in shake-flask level and crocetin was extracted and quantified according to the methods described below. The regression analysis was performed accordingly, which would show priority of all the tested factors (Supplementary Table S5). The significant factors were used for further optimization by steepest ascent method in which their concentration increased successively (Supplementary Table S6). The result was used to determine the central point of the following Box-Behnken response surface methodology (RSM) optimization (Supplementary Table S7) (Song et al., 2017).

The Design-Expert software was applied for the Box-Behnken Design, and three levels were selected for each factor (Supplementary Tables S7, S8). Through regression fitting analysis, a quadratic response surface regression model was established, according to which the optimal level of response factor for crocetin production was calculable.

### Fed-Batch Fermentation

The strain SyBE_Sc02130068 was used for fed-batch fermentation in 5-L bioreactor (BLBIO-5GJG-2, Shanghai, China). 100 μL glycerol-stock cell culture was inoculated into 25 mL SC-URA medium, culturing at 30°C, 250 rpm for overnight growth. Then the culture was transferred to 200 mL fresh SC medium and cultivated 10 h until OD_600_ reached about 5.0. Then, seed cultures were transferred to the bioreactor containing 1.8 L fermentation medium. The pH and the air flow were controlled at 5.5 and 2.5 vvm, respectively. And the dissolved oxygen was maintained above 40% through cascading with the stirring speed from 400 to 600 rpm. The temperature and feeding control varied according to the fermentation stage. The starting temperature of fermentation was 30°C, which was in favor of cell growth. As the crocetin pathway was controlled by GAL promoters (P*_GAL__1_*, P*_GAL__7_*, and P*_GAL__10_*), which were induced by galactose and inhibited by glucose. To get more cell density and higher yield, the fermentation process was divided into two stages: cell growth stage and crocetin accumulation stage. For the cell growth stage, fermentation was carried out at 30°C and 500 g/L concentrated glucose solution was feeding periodically to control the glucose concentration less than 1 g/L. 10 g yeast extract was added into the bioreactor every 12 h by adding 400 g/L concentrated yeast extract stock solution. When the cell grew into the plateau, the fermentation entered the crocetin accumulation stage. After the fermentation temperature was reduced to 20°C, 10 g/L of D-(+)-galactose was fed to induce crocetin formation. The feeding carbon source was changed into ethanol accordingly. The ethanol was controlled below 5 g/L through adjusting the feeding rate according to the ethanol concentration determined by HPLC.

### Carotenoids Extraction and Determination

The methods of carotenoids extraction and determination have been described previously (Chai et al., 2017). The harvested cells were broken through quick heating and cooling. The then carotenoids were extracted from the lysis solution (acetone containing 1% (w/v) butylated hydroxytoluene). The acetone phase was evaporated by nitrogen blow and then re-dissolved for HPLC analysis. To measure β-carotene and zeaxanthin, samples were dissolved in acetone and detected at 450 nm. In the meanwhile, for crocetin determination, sample was dissolved in methanol-dimethylformamide (7:1 v/v) and detected at 430 nm. Notably, brown centrifugal tubes were used during the whole procedure to avoid carotenoids degradation in light.

## Results and Discussion

### Bypath Disruption for Adequate Precursors

To increase the pools of the key precursor acetyl-CoA, some bypaths to convert acetyl-CoA into undesired products was attempted to be disrupted in our case. Krivoruchko et al. (2015) once reported that deletion of *CIT2* and *MLS1* enhanced 1-butanol production by improving acetyl-CoA supply *via* avoiding trapping acetyl-CoA GYC (Figure 1A). Accordingly, these two genes were deleted individually in our previous constructed crocetin producing strain SyBE_Sc0123C050. As shown in Supplementary Figure S1, individual deletion of these two genes was not resulted in growth defect under glucose. When glucose was exhausted and the carbon source was changed to ethanol, Δ*CIT2* and Δ*MLS1* were injurious to assimilation of ethanol to build biomass in different degrees. Chen et al. (2013) and Cobine et al. (2012) once described similar growth phenotypes of Δ*CIT2* and Δ*MLS1* on glucose and 2C carbon source (such as ethanol). Even though gene knockout decreased the final biomass, the total acetyl-CoA content as well as the crocetin yield per DCW(g) were both significantly enhanced by individual deletion of *CIT2* and *MLS1* (Figures 1B,C). The percent increase in the concentration of total acetyl-CoA corresponded with the enhancement in crocetin yield. Similar effect of Δ*CIT2* has also been observed on other terpene production (Cobine et al., 2012). Among these approaches, Δ*CIT2* achieved 49.7% improvement on total acetyl-CoA content and 1.5 times of enhancement on crocetin yield which was the higher one than *MLS1* knockout strains (Figure 1C). Therefore, *CIT2* deleted strain SyBE_Sc01010682 was employed as the candidate for further optimization.

### Facilitation of Catalysis Efficiency of CCD2 via Suited Fusion Protein

**FIGURE 2:**
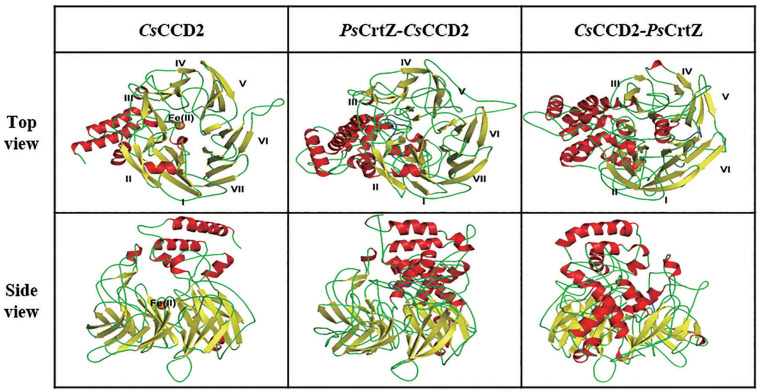
Tridimensional structure models of the *Cs*CCD*2* (left), forward fusion protein *Ps* CrtZ-*Cs*CCD*2* (middle) and reverse fusion protein *Cs*CCD*2-Ps*CrtZ (right). The ferrous catalytic iron is colored in orange. Secondary structural elements consisting of α-helices and β-sheets are colored in red and yellow. The blade numbers of the windmill structure are labeled.

Since CCDs catalyzed the limiting step for crocetin synthesis (Frusciante et al., 2014), fusion expression of *Ps*CrtZ and *Cs*CCD2 would facilitate the enzymic catalysis efficiency of CCD2 in terms of enhancing its substrate (zeaxanthin) accessibility. In previous studies, the fusion orientation has significantly affected protein structures and even the enzyme activities. Therefore, in our study, the structure models of native *Cs*CCD2, forward/reverse fusion of *Ps*CrtZ and *Cs*CCD2 were simulated and analyzed to guide our protein fusion design (Figure 2). The structural information of CCD2 showed that some non-contiguous α-helices and loops formed the hydrophobic substrate binding tunnel on the N-terminal and a highly conserved striking “windmill” architecture consisting of seven-bladed β-propeller formed the activity center on its C-terminal (Figure 2 left). The built model of forward fusion enzyme (*Ps*CrtZ-*Cs*CCD2) displayed the conserved windmill architecture with seven-blade (Figure 2 middle) which was consistent with the reported structure among CCDs family (Sui et al., 2013; Frusciante et al., 2014). Therefore, the enzymic activity of *Cs*CCD2 in forward fusion enzyme would be well retained. Moreover, the forward fusion of *Ps*CrtZ-*Cs*CCD2 shorted the distance between substrate (zeaxanthin) and N-terminal hydrophobic substrate binding tunnel of CCD2, providing an ideal environment for accumulation and availability of zeaxanthin substrates to *Cs*CCD2. On the contrary, in the built model of reverse fusion enzyme *Cs*CCD2-*Ps*CrtZ, some bladed β-propeller was replaced by the allochthonous α-helix, resulting in deletion of one of the original seven-blade (Figure 2 right). Therefore, the crucial windmill architecture of *Cs*CCD2 was disrupted by reverse fusion enzyme *Cs*CCD2-*Ps*CrtZ, which would lead to inactivation of *Cs*CCD2.

**FIGURE 3:**
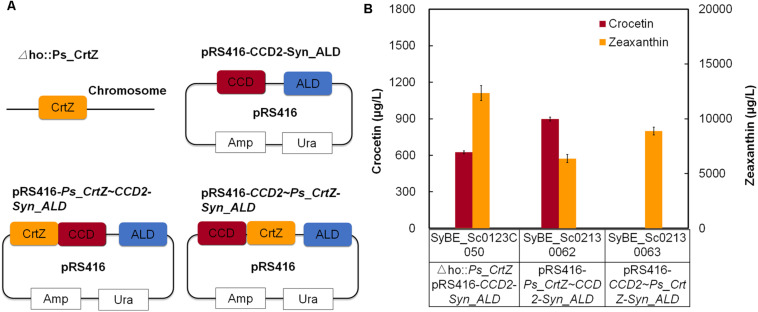
The effect of fusion orientation on crocetin production. **(A)** The construction of the expression modules; **(B)** The production of crocetin and zeaxanthin with related fusion proteins. The error bars represent standard deviations calculated from triplicate experiments.

Accordingly, the forward and reverse fusion of *Ps*CrtZ and *Cs*CCD2 (SyBE_Sc02130062 and SyBE_Sc02130063) were constructed (Figure 3A), and their effects on crocetin production were further investigated. As shown in Figure 3B, comparing with the crocetin titer in stain SyBE_Sc0123C050 with two unfused enzymes, the crocetin production was increased to 1.44 times by the forward fusion (*Ps*CrtZ-*Cs*CCD2), while no crocetin but an amount of zeaxanthin was detected in the strain SyBE_Sc01010685 with reverse fusion (*Cs*CCD2-*Ps*CrtZ). These results powerfully supported our hypothesis based on the structural analysis *in silico*. No conversion from zeaxanthin to crocetin suggested that *Cs*CCD2 in the reverse fusion enzyme *Cs*CCD2-*Ps*CrtZ was inactivated, which might due to the *Cs*CCD2 conformational change caused by reverse fusion version. Thus, this study is another successful case of linking up structural simulation with experimental test, which provided another reference to guide protein fusion except for our previous works (Jiang et al., 2017).

**FIGURE 4:**
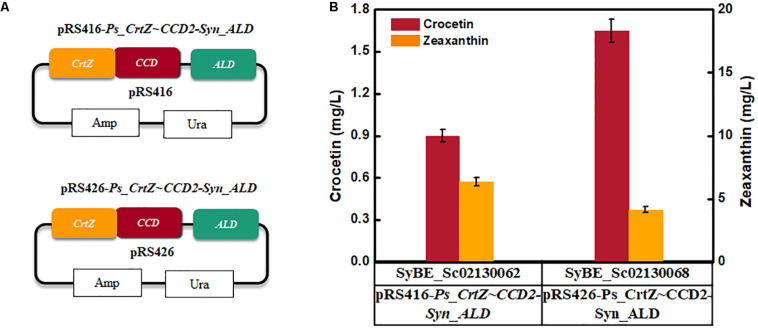
The effect of overexpression of fused CrtZ and CCD on crocetin titer. **(A)** The construction of the expression modules; **(B)** The production of crocetin and zeaxanthin with single-copy plasmid and multicopy plasmid, respectively. The error bars represent standard deviations calculated from triplicate experiments.

As the forward fusion protein *Ps*CrtZ-*Cs*CCD2 was proved to be more benefit for crocetin accumulation, thus its copy number was further raised *via* expressing the fusion protein by multicopy plasmid pRS426 instead of single-copy plasmid pRS416 (Figure 4A). As shown in Figure 4B, crocetin production was increased by 83% compared with the one achieved by single copy expression. Therefore, strain SyBE_Sc02130068 was used to process optimization.

### Crocetin Production Promoted by Medium Optimization

**FIGURE 5:**
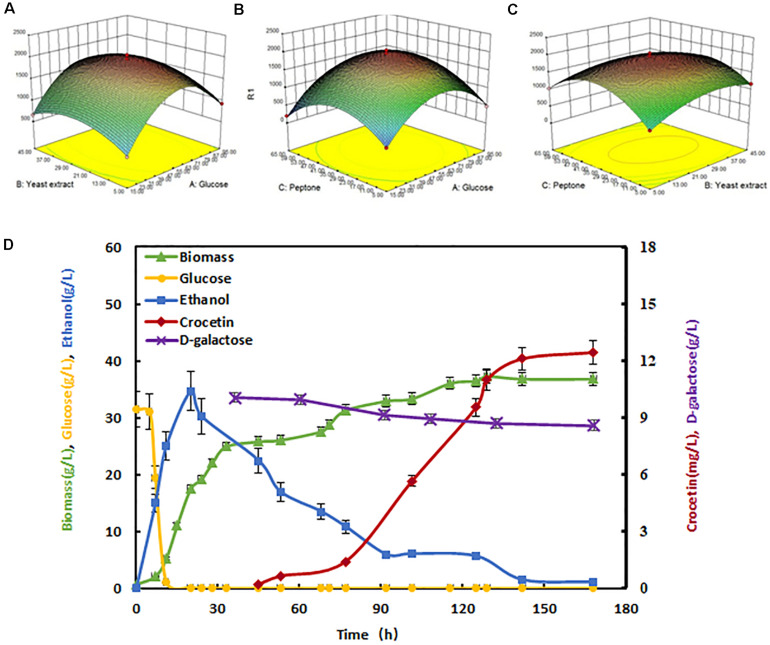
Crocetin production promoted by medium optimization and fed-batch fermentation. The 3D surface plots of each two factors’ interaction effect on the yield of crocetin. **(A)** The interaction effect of glucose and yeast extract on the crocetin yield; **(B)** The interaction effect of glucose and peptone on the crocetin yield; **(C)** The interaction effect of yeast extract and peptone on the crocetin yield; **(D)** Crocetin production in fed-batch fermentation. Crocetin production (red line), glucose (yellow line), ethanol (blue line), D-galactose (purple line), and biomass (green line) were measured during fed-batch fermentation. D-galactose was added at 36 h. The error bars represent standard deviation calculated from duplicate experiments.

Through pathway engineering and heterologous protein expression, especially after *CIT2* gene was knocked out, the growth has been significantly affected, indicating the endogenous metabolism flux of the engineered strain would change to some extent. In order to adapt to these changes, it is necessary to optimize the culture medium accordingly. Medium optimization was performed using the highest crocetin yield strain SyBE_Sc02130068. Firstly, Plackett-Burman (Ahuja et al., 2004) two-level fractional factorial design was used to verify the main effects among seven conventional ingredients (Supplementary Table S3). The regression analysis showed that the order of significance of the factor effects on crocetin production is: glucose = yeast extract > peptone > CaCl_2_ > KH_2_PO_4_ > (NH_4_)_2_SO_4_ (Supplementary Tables S4, S5). The main effect *P* values of glucose, yeast extract and peptone were less than 0.05, indicating that they were the main factors. Besides, these three factors were all have positive effects on output (*T* value is positive). Therefore, they were tested for further optimization by steepest ascent method and the results were shown in Supplementary Table S6. With the increase of the concentration of glucose, peptone, and yeast extract, the yield of crocetin was increased firstly and then decreased at certain points. As the yield of crocetin in the group 3 was the largest one, showing that the value of each factor in this group was close to the maximal response value region. Therefore, the factor value of the group 3 was taken as the central point of the follow-up Box-Behnken RSM optimization (Supplementary Table S7) (Song et al., 2017). The experimental results were listed in Supplementary Table S8. The 3D surface plots of each two factors’ interaction effect on the yield of crocetin were shown in Figures 5A–C. Through regression fitting analysis, a quadratic response surface regression model was established, and the fitting quadratic regression equation was obtained as belows:

R=1555.83462+62.78675⁢A+53.76462⁢B+56.78296⁢C

(1) -0.032852⁢A⁢B+0.051083⁢A⁢C-0.25913⁢B⁢C-0.54415⁢A⁢2-0.81730⁢B⁢2-0.76603⁢C⁢2

In this equation, R, A, B, and C represented crocetin production, glucose concentration, yeast extract concentration, and peptone concentration, respectively. Through this equation, the optimal medium formulation was obtained with 58.54 g/L glucose, 26.24 g/L yeast extract, 34.56 g/L peptone, and the theoretical prediction of the maximum yield was 1.97 mg/L accordingly. The verification experiment was also done with this predicted concentration of medium ingredients, and the actual crocetin production was 1.95 ± 0.23 mg/L, which matched the prediction model very well. The correctness between the prediction and experimental results verified the credibility of the quadratic response surface regression model. The crocetin titer with the optimized fermentation medium increased by 18.5% compared to the traditional YPD medium.

### Crocetin Output Further Enhanced by Fed-Batch Fermentation

To further promote the crocetin production, the engineered strain SyBE_Sc02130068 was selected for fed-batch fermentation in 5-L fermentor using the optimized medium above. As shown in Figure 5D, the fermentation process was divided into two stages. The first stage was cell growth stage in which the cultivation temperature was controlled at 30°C for 35 h until the cell density OD_600_ reached 105. The supplemental carbon source glucose was strictly controlled below 1 g/L due to carbon source restriction strategy. The second stage crocetin producing stage, was initialized after the temperature was decreased to 20°C and 10 g/L D-(+)-galactose was added at 36 h. The supplemental carbon source was changed into ethanol, because the existence of glucose would inhibit the expression of the GAL promoters. The ethanol concentration was maintained at nearly 5 g/L until fermentation end. After 70 h, the crocetin production increased exponentially until 124 h. It was also noticed that while the crocetin accumulated sharply, the cell growth did not increase significantly. Thus, increasing the crocetin resistance in yeast will be a promising solution for crocetin further improvement. After 160 h cultivation, the crocetin titer reached 12.43 ± 0.62 mg/L (Figure 5D), which was eight times more than the output in shake flask level. As shown in Figure 5D, it is found that galactose concentration decreased with increasing concentration of crocetin. And the galactose consumption might affect the production of crocetin. The absolute titer (12.43 mg/L) and the production yield based on ethanol consumption (*Y*_P/S_ = 0.017%) were still far away from commercialization level, strain development by metabolic engineering and synthetic biology to increase the product accumulation rate and product resistance, as well as process optimization for higher cell density fermentation would be basic and efficient solutions. In addition, together with off-gas analysis, involving more on-line parameters associated with growth and production as feedback will also be helpful for precise control in fermentation (Sui et al., 2016), leading to higher crocetin production.

## Conclusion

Further enhancing the accumulation of natural products with complex structure in microbes is always a big challenge. Here, a systematic optimization was executed for crocetin overproduction in *S. cerevisiae*, mainly based on promoting precursor pools, tuning key enzymes as well as optimizing bioprocess. Through above optimizations, the crocetin production in final strain SyBE_Sc02130068 was stepwise increased by 212% compared to that in the initial strain SyBE_Sc0123C050 at shake-flask level. Finally, a titer of 12.43 ± 0.62 mg/L crocetin was realized by 5-L fed-batch fermentation, which was the highest crocetin titer reported in micro-organisms to date. This study not only set a good example to guide audience how to stepwise increase heterologous natural products accumulated in microbials, but also highlights the synergy between precursor engineering and key enzymes engineering is crucial for obtaining microbial overproduction of natural products.

## Data Availability Statement

All datasets presented in this study are included in the article/Supplementary Material.

## Author Contributions

TS and MY conceived the study. NW and CW participated in strain construction. MY and NW carried out the protein analysis. TS and WX participated in fed-batch fermentation. TS, CW, and FC carried out the chemical analysis. MD, YW, and XL helped to draft the manuscript. YY participated in design and coordination of the study. MY supervised the whole research and revised the manuscript. All the authors read and approved the final manuscript.

## Conflict of Interest

The authors declare that the research was conducted in the absence of any commercial or financial relationships that could be construed as a potential conflict of interest.
