# Supplementary material for: Crocetin Overproduction in Engineered Saccharomyces cerevisiae via Tuning Key Enzymes Coupled With Precursor Engineering
Source: Front Bioeng Biotechnol. 2020 Sep 4;8:578005. doi: 10.3389/fbioe.2020.578005 (PMC7500066; doi:10.3389/fbioe.2020.578005)
Supplement: Supplementary file 1 [file Table_1.DOCX]

**Supporting information**

Crocetin overproduction in engineered *Saccharomyces cerevisiae* via tuning key enzymes coupled with precursor engineering

**Tianqing Song^1,2^,** **Nan Wu^1,2^, Chen Wang^1,2^, Ying Wang^1,2^, Fenghua Chai^1,2^, Mingzhu Ding^1,2^, Xia Li^1,2^, Mingdong Yao^1,2*^, Wenhai Xiao^1,2^, Yingjin Yuan^1,2^**

^1^ Frontier Science Center for Synthetic Biology and Key Laboratory of Systems Bioengineering (Ministry of Education), School of Chemical Engineering and Technology, Tianjin University, Tianjin 300072, China

^2^ Collaborative Innovation Center of Chemical Science and Engineering (Tianjin), Tianjin University, Tianjin 300072, China

***Correspondence:**
Mingdong Yao
Email: mingdong.yao@tju.edu.cn

**Table S1. Plasmids and primers used in this study**

| **Plasmid** | **Description** | **Source** |
| --- | --- | --- |
| pUC57-Simple-05 | Plasmid harboring *CrtZ* from *Pantoea stewartii* (*PsCrtZ*) | This Lab |
| pUC57-Simple-10 | Plasmid harboring *CCD2* from *Crocus* (*CsCCD2*) | This Lab |
| pUC57-Simple-16 | Plasmid harboring *ALD* from *Synechocystis* sp. PCC6803 (*SynALD*) | This Lab |
| pEASY-Blunt-Q-03 | Plasmid harboring *CIT2* knockout cassette (pEASY blunt-*CIT2*_L-*URA3*-*CIT2*_R) | This study |
| pEASY-Blunt-Q-04 | Plasmid harboring *MLS1* knockout cassette (pEASY blunt-*MLS1*_L-*URA3*-*MLS1*_R) | This study |
| pRS416-A-04 | pRS416 harboring the cassette used for expression of *CsCCD2* and *SynALD* (T*_HIS5_-*P*_GAL10_-CsCCD2-*T*_TEF2_* *-*P*_GAL7_-*Syn*ALD-*T*_PGI1_*) | This study |
| pRS416-L-04 | pRS416 harboring the cassette used for co-expression of *PsCrtZ* and *CsCCD2* in the forward fusion version (P*_GAL10_*-*PsCrtZ****--****CsCCD2*-T*_TEF2_*-P*_GAL7_*-*SynALD*-T*_PGI1_*) | This study |
| pRS416-L-05 | pRS416 harboring the cassette used for co-expression of *PsCrtZ* and *CsCCD2* in the reverse fusion version (P*_GAL10_*-*CsCCD2****--****PsCrtZ* -T*_TEF2_*-P*_GAL7_*-*SynALD*-T*_PGI1_*) | This study |
| pRS426-L-01 | pRS426 harboring the cassette used for co-expression of *PsCrtZ* and *CsCCD2* in the forward fusion version (P*_GAL10_*-*PsCrtZ*--*CsCCD2*-T*_TEF2_*-P*_GAL7_*-*SynALD*-T*_PGI1_*) | This study |

**Table S2. Primers used in this study**

| **Primer** | **Description** |
| --- | --- |
| **For construction of cassette *CIT2*_L-*URA3*-*CIT2*_R** | |
| CIT2_L-F^a^ | *GGAATTCCATATG* GTAGCTAGACGTCTATCAGG |
| CIT2_L-R^b^ | GGGAATTGCCATGAAGCCGAATTTTCTTGTTACTAGTATTATTAAAA |
| URA3-F^b^ | AATAATACTAGTAACAAGAAAA TTCGGCTTCATGGCAAT |
| URA3-R^b^ | GTAAAAGTAGGATGTAATCCAA GGTAACGCCAGGGTTTTC |
| CIT2_L-F^b^ | GGGAAAACCCTGGCGTTACC TTGGATTACATCCTACTTTTAC |
| CIT2_L-R^a^ | *GGAATTCCATATG* AGGTTCTTAATAAGTTATTACTAAA |
| **For construction of cassette *MLS1*_L-*URA3*-*MLS1*_R** | |
| MLS1_L-F^a^ | *GGAATTCCATATG* TGCAGTGTCAGCCTTAC |
| MLS1_L-R^b^ | GGGAATTGCCATGAAGCCGAA TTTCTTAATTCTTTTATGTGC |
| URA3-F^b^ | AAGCACATAAAAGAATTAAGAAA TTCGGCTTCATGGCAAT |
| URA3-R^b^ | GTACACTGGGGCAAGGGAGA GGTAACGCCAGGGTTTTC |
| MLS1_L-F^b^ | GGGAAAACCCTGGCGTTACC TCTCCCTTGCCCCAGT |
| MLS1_L-R^a^ | *GGAATTCCATATG* GCTGAAAGCTTCAAACGTATA |

^a^ Restriction site was in italic.

^b^ Homologous overhang-nucleotides were underlined

**Table S3. Plackett-Burman design**

| **Factors** | **Levels** | |
| --- | --- | --- |
|  | -1 | 1 |
| Glucose | 15 | 55 |
| Yeast extract | 5 | 20 |
| Peptone | 5 | 30 |
| (NH_4_)_2_SO_4_ | 0 | 2 |
| MgSO_4_ | 0 | 5 |
| KH_2_PO_4_ | 0 | 5 |
| CaCl_2_ | 0 | 0.5 |

**Table S4. Plackett-Burman results**

| No. | Glucose  (g/L) | Yeast extract  (g/L) | Peptone  (g/L) | (NH_4_)_2_SO_4_  (g/L) | MgSO_4_  (g/L) | KH_2_PO_4_  (g/L) | CaCl_2_  (g/L) | Crocetin  (μg/L) |
| --- | --- | --- | --- | --- | --- | --- | --- | --- |
| 1 | 55 | 5 | 30 | 0 | 5 | 0 | 0 | 1239 |
| 2 | 15 | 5 | 5 | 0 | 0 | 0 | 0 | 515 |
| 3 | 15 | 20 | 30 | 0 | 0 | 5 | 0 | 1367 |
| 4 | 15 | 20 | 30 | 2 | 5 | 0 | 0.5 | 1439 |
| 5 | 15 | 5 | 30 | 0 | 5 | 5 | 0.5 | 902 |
| 6 | 55 | 5 | 30 | 2 | 0 | 0 | 0.5 | 1576 |
| 7 | 55 | 20 | 5 | 0 | 0 | 0 | 0.5 | 1803 |
| 8 | 15 | 5 | 5 | 2 | 0 | 5 | 0.5 | 554 |
| 9 | 55 | 5 | 5 | 2 | 5 | 5 | 0 | 1124 |
| 10 | 55 | 20 | 5 | 0 | 5 | 5 | 0.5 | 1565 |
| 11 | 15 | 20 | 5 | 2 | 5 | 0 | 0 | 1025 |
| 12 | 55 | 20 | 30 | 2 | 0 | 5 | 0 | 1916 |

**Table** **S5. Regression analysis of Plackett-Burman**

| **Factors** | **Levels** | | |
| --- | --- | --- | --- |
|  | T | P | significance |
| Glucose (g/L) | 8.40 | 0.001 | 1 |
| Yeast extract (g/L) | 7.92 | 0.001 | 1 |
| Peptone (g/L) | 4.95 | 0.008 | 2 |
| (NH_4_)_2_SO_4_ (g/L) | 0.56 | 0.607 | 5 |
| MgSO_4_ (g/L) | 0.51 | 0.637 | 6 |
| KH_2_PO_4_ (g/L) | 1.41 | 0.230 | 4 |
| CaCl_2_ (g/L) | -1.84 | 0.140 | 3 |

**Table S6. Steepest ascent design**

|  | Glucose (g/L) | Yeast extract (g/L) | Peptone (g/L) | Crocetin(μg/L) |
| --- | --- | --- | --- | --- |
| 1 | 15 | 5 | 5 | 355 |
| 2 | 35 | 15 | 20 | 939 |
| 3 | 55 | 25 | 35 | 1994 |
| 4 | 75 | 35 | 50 | 1040 |
| 5 | 95 | 45 | 65 | 529 |

**Table S7. Box-Behnken design**

| **Factors** | **Levels** | | |
| --- | --- | --- | --- |
|  | -1 | 0 | 1 |
| Glucose (g/L) | 15 | 55 | 95 |
| Yeast extract (g/L) | 5 | 25 | 45 |
| Peptone (g/L) | 5 | 35 | 65 |

**Table S8. Box-Behnken results**

|  | Glucose (g/L) | Yeast extract (g/L) | Peptone (g/L) | Crocetin(μg/L) |
| --- | --- | --- | --- | --- |
| 1 | 55 | 45 | 65 | 802 |
| 2 | 15 | 45 | 35 | 652 |
| 3 | 55 | 25 | 35 | 1945 |
| 4 | 55 | 25 | 35 | 1972 |
| 5 | 95 | 45 | 35 | 954 |
| 6 | 15 | 25 | 5 | 331 |
| 7 | 95 | 5 | 35 | 925 |
| 8 | 55 | 45 | 5 | 1166 |
| 9 | 95 | 25 | 65 | 592 |
| 10 | 15 | 25 | 65 | 200 |
| 11 | 15 | 5 | 35 | 518 |
| 12 | 55 | 5 | 5 | 774 |
| 13 | 55 | 25 | 35 | 2057 |
| 14 | 55 | 25 | 35 | 1924 |
| 15 | 55 | 5 | 65 | 1033 |
| 16 | 95 | 25 | 5 | 478 |
| 17 | 55 | 25 | 35 | 1902 |

**Table S9 Reaction Mixes**

Assay Range(0-1nmole)

| Reagent | Samples and  Standars/μL | Blank Sample/μL |
| --- | --- | --- |
| Acetyl-CoA Assay Buffer | 40 | 41 |
| Acetyl-CoA Substrate Mix | 2 | 2 |
| Conversion Enzyme | 1 | - |
| Acetyl-CoA Enzyme Mix | 5 | 5 |
| Fluorescent Probe | 2 | 2 |

Assay Range(0-100 pmole)

| Reagent | Samples and  Standars/μL | Blank Sample/μL |
| --- | --- | --- |
| Acetyl-CoA Assay Buffer | 41.8 | 42.8 |
| Acetyl-CoA Substrate Mix | 2 | 2 |
| Conversion Enzyme | 1 | - |
| Acetyl-CoA Enzyme Mix | 5 | 5 |
| Fluorescent Probe | 0.2 | 0.2 |


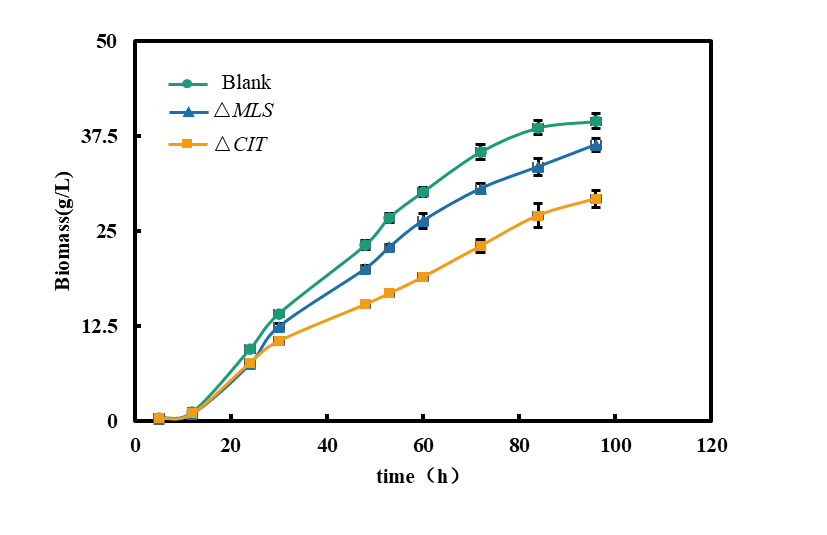


**Figure S1. Effect of gene deletion on cell growth.** The control and gene knock-out strains (*△CIT2* and *△MLS1*) were cultured in shake flask with YPD medium containing 20 g/L glucose. The arrow indicated the time that the glucose was depleted. All measurements are means of three independent biological replicates.
